# Associations between prevalent unhealthy lifestyles and the gut microbiota: a comprehensive multi-database bibliometric analysis of pathogenic mechanisms and clinical trajectories

**DOI:** 10.3389/fmed.2026.1834916

**Published:** 2026-05-12

**Authors:** Xiao-Jie Zhou, Jun-Lei Chen, Chen-Feng Hu, Zhang-Kai Li, Hong-Bo Zheng, Tu-Nan Ding, Yan Chen, Yan Zhu, Jia-Ni Li, Qiang Fu, Yin Fu

**Affiliations:** 1 First Clinical Medical College, Heilongjiang University of Chinese Medicine, Harbin, Heilongjiang, China; 2 School of Traditional Chinese Medicine, Beijing University of Chinese Medicine, Beijing, China; 3 Department of Gastroenterology, Beijing Hospital of Integrated Traditional Chinese and Western Medicine, Beijing, China; 4 Department of General Surgery, The Second Affiliated Hospital of Fujian University of Traditional Chinese Medicine, Fuzhou, Fujian, China; 5 Department of Basic Medical Science, Heilongjiang University of Chinese Medicine, Harbin, Heilongjiang, China

**Keywords:** bibliometric analysis, clinical translation, gastrointestinal microbiome, inflammation, metabolic dysfunction, obesity, unhealthy lifestyle

## Abstract

**Background:**

Unhealthy lifestyles are associated with gut microbiota dysbiosis through complex inflammatory and immune-related pathways. Despite extensive primary research, comprehensive big-data syntheses mapping this field remain limited. This study systematically analyzes the research landscape, related pathophysiological processes, and emerging trends in the field of lifestyle–microbiome interactions using a multi-database bibliometric framework.

**Methods:**

A dual-database retrieval strategy utilized the Web of Science Core Collection (WoSCC) and PubMed. The analysis targeted five predominant unhealthy lifestyles: smoking, alcohol consumption, sleep disorders, sedentary behavior, and high-sugar diets. A primary dataset of 5,380 records published between January 1, 2001, and March 13, 2026, was extracted from WoSCC. A parallel clinical analysis incorporated 172 targeted studies from PubMed. Bibliometric analyses, encompassing publication trends, network topologies, citation bursts, keyword evolution, and clinical trajectories, were performed and visualized using Microsoft Excel 2024, VOSviewer, CiteSpace, SCImago, and R.

**Results:**

Publication output exhibited exponential growth from 2001 to 2026. China and the United States emerged as the dominant academic contributors. Thematic analyses identified inflammation, oxidative stress, obesity, metabolic dysfunction, and microbiome-associated metabolomics as core pathophysiological nodes. The literature frequently describes these elements as mediating the association between behavioral exposures and microbial dysbiosis. Clinical research trajectories demonstrated an evolution toward high-rigor methodological designs, systemic blood biomarker integration, and demographic stratification. The bibliometric data highlight targeted lifestyle intervention as a heavily researched non-pharmacological strategy for gut microbiota preservation.

**Conclusion:**

This multi-database bibliometric study delineates the structural landscape of research on the association between unhealthy lifestyles and the gut microbiota. Inflammation, oxidative stress, and metabolic dysfunction emerge as central themes within the existing literature. These findings provide a systematic framework for understanding microenvironmental interactions and offer valuable insights to inform future research and the development of precise, lifestyle-oriented strategies for gastrointestinal health.

## 1 Introduction

The human gut microbiota centrally regulates host health (1). Gut dysbiosis functions as a critical mechanistic bridge. It translates environmental exposures into the pathogenesis of chronic diseases, inflammatory bowel disease, and cardiovascular disorders (2). Consequently, identifying modifiable lifestyle drivers of this dysbiosis represents a global translational and public health priority (3).

Modern industrialization actively drives the global proliferation of unhealthy lifestyles (4). Fast-paced societal structures systematically exacerbate maladaptive behavioral patterns (5). Among various unhealthy behaviors, tobacco smoking, alcohol consumption, sleep disorders, high-sugar diets, and sedentary behavior stand out as highly prevalent, representative, and modifiable risk factors, which are also highlighted in the Life’s Essential 8 (LE8) framework (6). Recent epidemiological evidence identifies these five variables as key factors associated with gut microbiota dysbiosis (7). These distinct factors collectively account for the vast majority of preventable global morbidity (8). According to the Global Burden of Disease (GBD) Study, tobacco smoking remains a leading preventable cause of morbidity and mortality. It is responsible for approximately 8.7 million deaths and over 229 million disability adjusted life years (DALYs) annually worldwide (9). The 2024 World Health Organization Global Status Report further identifies alcohol consumption as a critical global health threat. It causes 2.6 million deaths annually and accounts for 4.7% of all deaths worldwide (10). In addition, GBD data indicate that high fasting plasma glucose, largely resulting from high sugar diets, causes approximately 6.5 million deaths and 172 million DALYs globally (9). This underscores its profound metabolic and oncologic consequences. Sleep disturbances represent another major behavioral risk factor. Extensive epidemiological evidence confirms that sleep disorders have emerged as a critical public health challenge. This builds on the landmark discoveries of circadian rhythm mechanisms recognized by the 2017 Nobel Prize in Physiology or Medicine. Sleep disorders affect an estimated 30 to 35% of the global adult population. Nearly 10% suffer from clinical chronic insomnia (11). Circadian disruption contributes to immune dysregulation, oxidative stress, and metabolic imbalance. Furthermore, prolonged sedentary behavior is widespread. It currently affects approximately 27.5% of adults globally (12). It increases all-cause mortality risk by 20 to 30% and contributes to over 3.2 million preventable deaths annually (13). Crucially, these five behaviors frequently co occur in industrialized populations. They form complex synergistic pathogenic networks. They exert severe synergistic effects and establish a persistent cycle between behavioral exposures and microbial imbalance (14, 15). To ensure strong epidemiological relevance and translational value, this study focuses on these five predominant and modifiable health behaviors.

Advanced multi-omics and bioinformatics continuously expand this research domain (16). However, current knowledge remains highly fragmented. Most investigations isolate single lifestyle factors (17). A systematic, multi-database synthesis encompassing these five core interconnected behaviors is currently absent.

This study addresses this critical knowledge gap. We integrated comprehensive data from multiple databases and conducted an advanced bibliometric analysis. Previous bibliometric studies have broadly explored the gut microbiome. However, our study provides a highly targeted and updated macroscopic overview (18). We specifically focus on the intersection and combined impact of these five prevalent and highly representative unhealthy behaviors on the gut microbiota. By delineating temporal trajectories, identifying core hotspots, and mapping collaborative networks, we define the structural knowledge architecture of the field. These insights establish precise priorities for future mechanistic research. Ultimately, this multi-database mapping provides evidence-based, translational guidance for lifestyle-targeted public health interventions.

## 2 Materials and methods

### 2.1 Data collection

The concept of “unhealthy behaviors” is broad and generates a massive body of literature. A generalized search would create highly cluttered data. To minimize this analytical noise, we conducted a preliminary search guided by the LE8 framework (6). By integrating this framework with current GBD epidemiological data, we identified five universally recognized lifestyle factors. These are tobacco smoking, alcohol consumption, sleep disturbances, high-sugar diets, and prolonged sedentary behavior. The literature volume for these specific variables is highly moderate. It provides a robust dataset but strictly avoids being overly vast or fragmented. This makes it ideal for a focused bibliometric analysis. Furthermore, authoritative data robustly support this selection. The GBD 2021 study and WHO reports explicitly identify these five exposures as leading drivers of preventable global mortality (8, 10).

To ensure methodological rigor, we adopted a strict dual database parallel design. The Web of Science Core Collection (WoSCC) served as the primary source for macroscopic bibliometric mapping. PubMed acted as an independent supplementary database for clinical research analysis. We analyzed datasets from WoSCC and PubMed separately to prevent structural artifacts from database incompatibility (19). The records were never merged across databases at any stage of data processing, network construction, or visualization (20). Literature was systematically retrieved from the Web of Science Core Collection and PubMed on March 13, 2026. The TS (Topic) field was used in the Web of Science Core Collection. The [tiab] (Title/Abstract) field was applied in PubMed. All search terms were standardized according to Medical Subject Headings (21). We conducted five independent searches within each database. Each search paired one specific lifestyle behavior with gut microbiota terms. This method ensured comprehensive coverage. It enabled precise tracking of subtheme publication trends. It also facilitated a focused analysis of shared keywords linking the five unhealthy behaviors with the gut microbiota. The complete search strategies are provided in Supplementary Table 1.

Strict inclusion and exclusion criteria were applied to the retrieved records. We limited the final inclusion strictly to English language publications. This restriction guarantees uniform literature quality (22). It also crucially prevents execution errors in the bibliometric software during subsequent analysis (23). Regarding document types, the WoSCC dataset was restricted to peer reviewed original articles and reviews. The PubMed dataset was exclusively restricted to clinical study types as detailed in Supplementary Table 1. Data integration and cleaning were performed using the R package bibliometrix (version 5.2.1). The five distinct datasets within each database were combined. We then conducted rigorous deduplication using Digital Object Identifiers. Individual studies frequently address multiple lifestyle factors simultaneously. Deduplication using DOIs effectively prevented double counting and ensured absolute dataset accuracy. Records lacking essential metadata were ultimately excluded. Two independent investigators performed the literature screening. A third senior investigator resolved any discrepancies.

**Figure 1:**
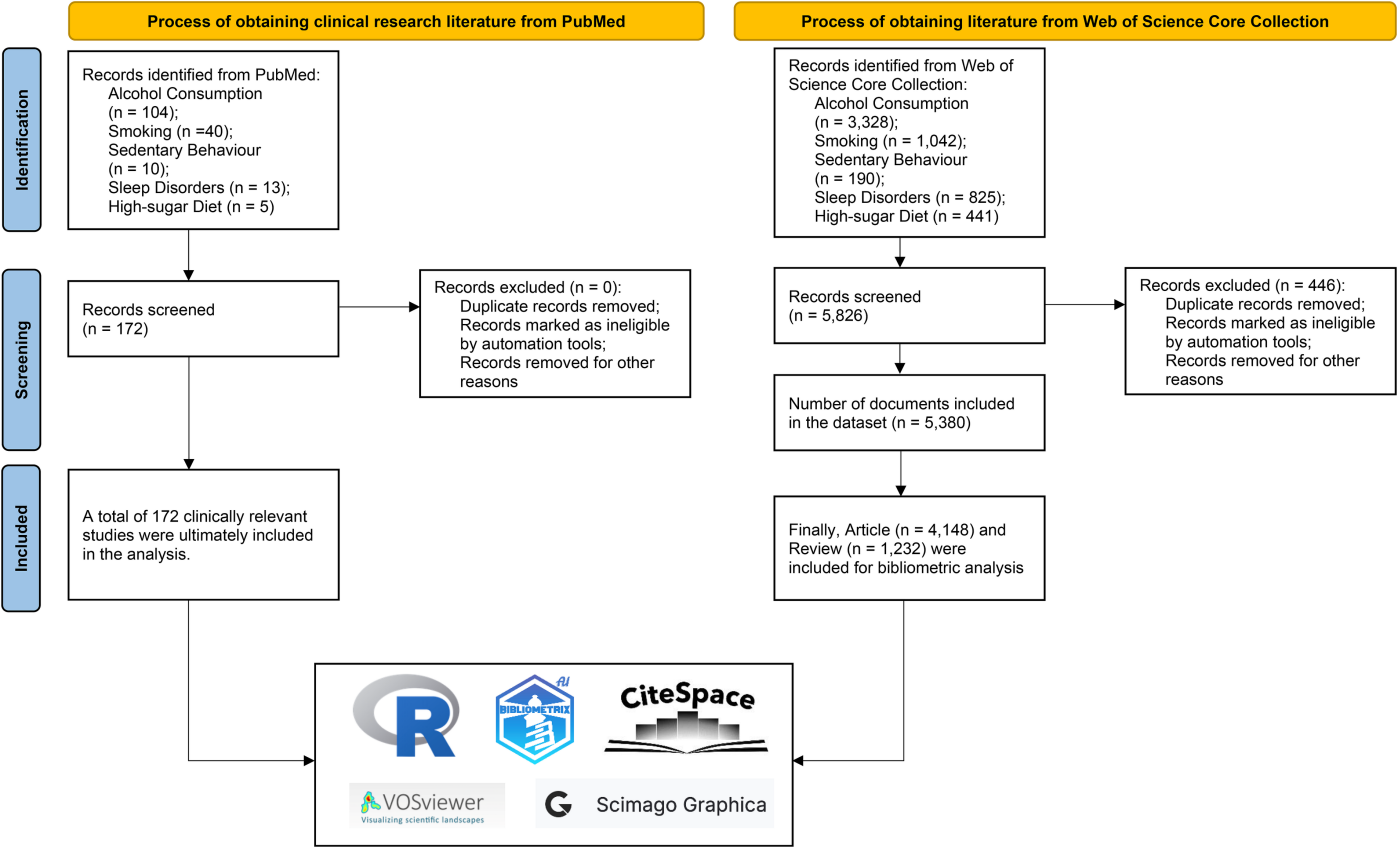
PRISMA-adapted flow diagram of the literature retrieval and screening process.

The final WoSCC dataset comprised 5,380 articles spanning 1,389 journals (Supplementary Figure 1). The parallel PubMed search yielded 172 clinical studies across 105 journals (Supplementary Figure 2). Journal Impact Factor and Journal Citation Reports (JCR) quartiles were retrieved from the 2024 JCR release. To ensure methodological transparency, all retrieval and screening procedures were conducted using a PRISMA inspired framework adapted for bibliometric analysis. Unlike traditional systematic reviews and meta-analyses, bibliometric studies focus on the quantitative analysis of large-scale literature datasets and do not involve risk of bias assessment (19). The complete step by step workflow is presented in Figure 1. To ensure methodological rigor, this study was conducted and reported in strict accordance with the BIBLIO checklist for reporting bibliometric reviews of the biomedical literature (Supplementary Table 2) (24).

### 2.2 Data analysis

Bibliometric analysis and visualization were conducted using several specialized computational tools (23, 25). First, R (version 4.5.2) with the bibliometrix package (version 5.2.1) extracted the primary data. Extracted metrics included overall annual publication trends, sub-dataset publication trends, corresponding author countries, total national outputs, and keyword frequencies. This extraction covered both the overall dataset and the five sub-datasets. The bibliometrix package also generated author annual production maps, thematic evolution plots, and thematic conceptual maps. Next, VOSviewer (version 1.6.20) was used for co-occurrence analyses. It constructed network visualization maps for institutions, journals, authors, and keywords. Additionally, CiteSpace (version 6.4. R1) performed citation burst analysis using default parameters. Microsoft Excel 2024 managed general data and plotted the overall annual publication trends. For advanced presentation, the R package ggplot2 visualized data derived from bibliometrix and VOSviewer. Specifically, ggplot2 created sub-theme annual trend lines, country radar charts, and institutional lollipop plots. It also generated dual-metric bubble charts (displaying both publications and citations) for journals and authors, alongside heatmaps for keywords and sub-theme intersections. Finally, SCImago Graphica (beta 1.0.53) mapped national geographical contributions and constructed institutional chord diagrams. All software tools were accessed via legitimate institutional licenses.

Prior to network visualization, targeted data preprocessing was conducted to ensure analytical accuracy and reproducibility. Institutional affiliations were manually screened and standardized to merge variant expressions referring to the same entities, thereby reducing fragmentation in institutional data. Journal names were also harmonized to resolve inconsistencies in naming conventions. For keyword processing, a selective normalization strategy was applied. General and structurally redundant terms were standardized where appropriate. However, for core conceptual terminology related to the study focus, a conservative strategy was adopted. Specifically, the terms “gut microbiota,” “microbiome,” and “microbiota” were intentionally retained as independent keywords rather than being merged. This decision was made to preserve terminological heterogeneity in the literature and to enable the tracking of temporal shifts in scientific language usage across the study period.

## 3 Results

### 3.1 Overview of themes and sub-themes

**Figure 2:**
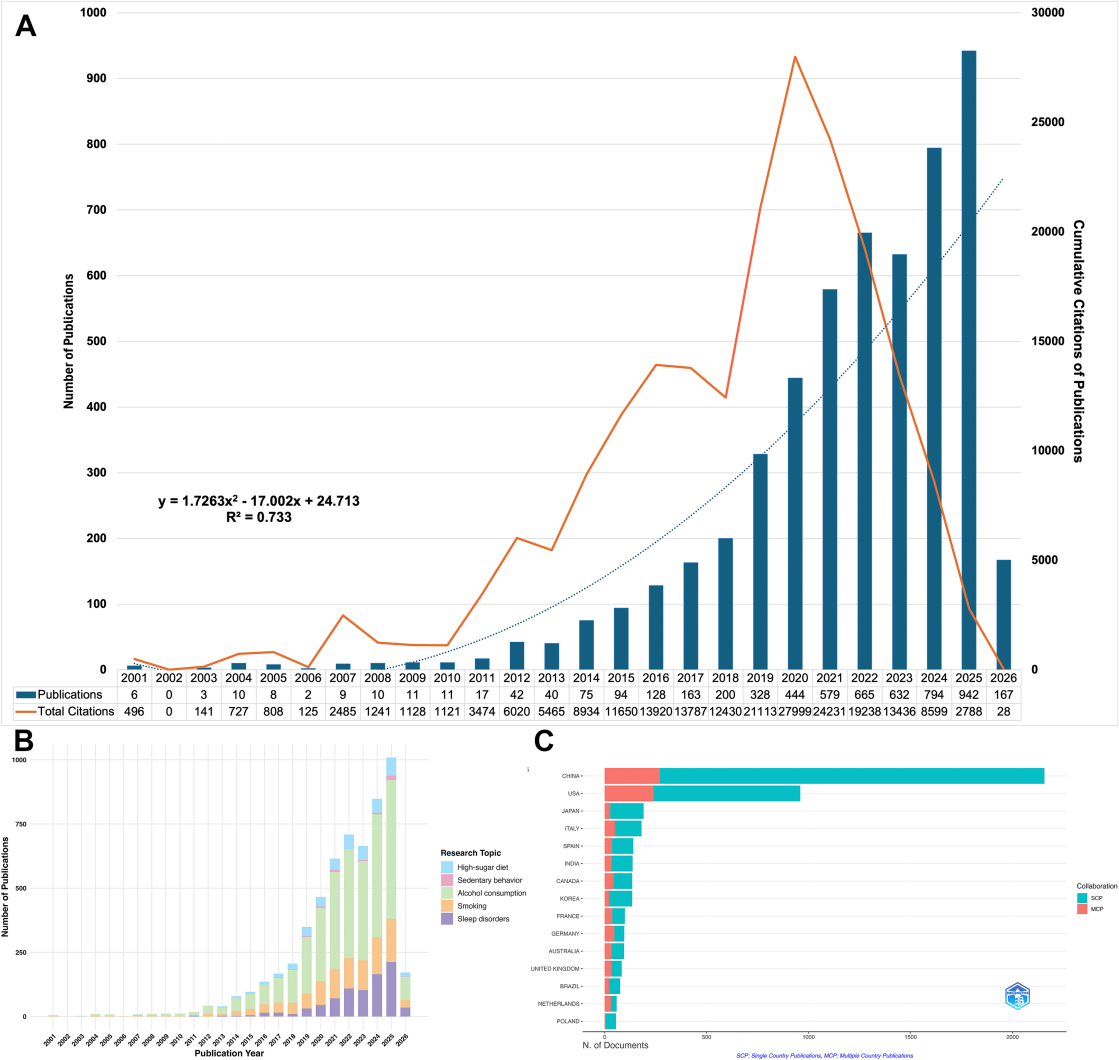
Analysis of publications from 2001 to 2026. **(A)** Annual publication trends, cumulative citations of publications, and polynomial fitting curves regarding the impact of unhealthy lifestyles on the gut microbiota. **(B)** Annual distribution of publication volumes across the five behavioral sub-themes. **(C)** Geographic distribution of corresponding authors.

The main WoSCC dataset comprised 5,380 publications. This total consisted of 4,148 articles and 1,232 reviews. As shown in Figure 2A, annual publications in this field increased from 6 in 2001 to 942 in 2025. The average annual growth rate was 37.44%. The growth curve exhibited a strong fit (R^2^ = 0.733). Publication output occurred in three distinct phases. The initial phase (2001–2011) showed slow, fluctuating growth. Annual outputs increased from 6 to 17, with a 1% average growth rate. The second phase (2012–2018) demonstrated steady growth. Publications rose from 42 to 200, averaging a 22.57% growth rate. The third phase (2019–2025) marked a period of rapid explosion. Annual outputs surged from 328 to 942, with a high average growth rate of 87.71%. Data collection for 2026 was restricted to a three-month period. Despite this partial dataset, the fitted trend line projects continued significant growth for the full year. Cumulative citation analysis revealed a peak in 2020. Articles published in this year received the highest number of citations (TC = 27,999). More recent publications showed predictably lower citation counts. This decrease simply reflects the inherent time lag in citation accumulation.

Figure 2B details the annual publication trends for specific unhealthy lifestyle factors. Alcohol consumption consistently dominated the research output. Conversely, sedentary behavior accounted for the fewest publications.

Figure 2C and Table 1 summarize the national distribution of corresponding authors. China contributed the highest number of publications (NP = 2,156). The United States (NP = 959) and Japan (NP = 164) followed. However, a stark contrast was observed in China’s research output between single-country publications (SCP = 1,886) and multiple-country publications (MCP = 270). This large disparity indicates a strong domestic research focus and a notable lack of international collaboration.

**Table 1:** Top 10 most productive countries and regions of corresponding authors.

Rank	Country	NP	NP %	SCP	MCP	MCP %
1	CHINA	2,156	40.1	1886	270	12.5
2	USA	959	17.8	720	239	24.9
3	JAPAN	191	3.6	164	27	14.1
4	ITALY	181	3.4	131	50	27.6
5	SPAIN	140	2.6	104	36	25.7
6	INDIA	137	2.5	104	33	24.1
7	CANADA	135	2.5	93	42	31.1
8	KOREA	135	2.5	114	21	15.6
9	FRANCE	99	1.8	62	37	37.4
10	GERMANY	96	1.8	49	47	49

NP, number of publications; SCP, single-country publications; MCP, multiple-country publications (international collaborations); MCP Ratio = MCP/NP.

### 3.2 Country analysis

**Figure 3:**
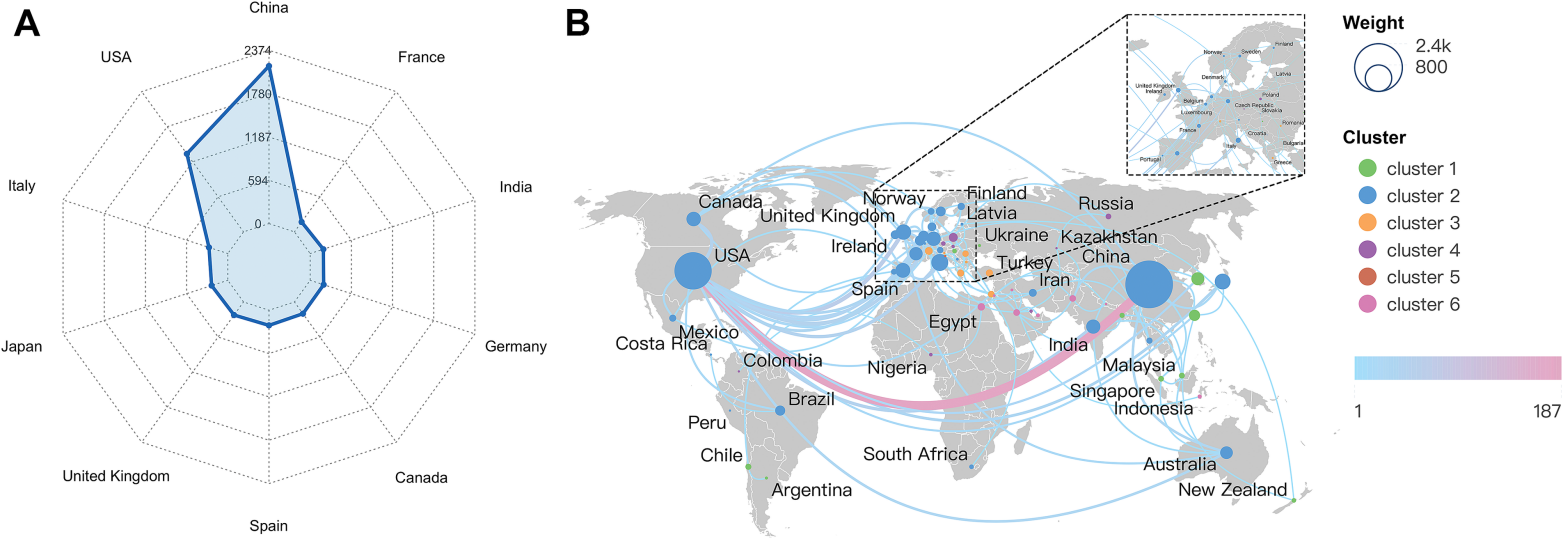
National and regional collaboration analysis. **(A)** Top productive countries and regions. **(B)** Geographic visualization of national collaboration and publication output.

Figure 3A illustrates the distribution of national publication volumes. China and the United States maintained an overwhelming lead. Figure 3B maps the geographical network of international collaborations. The strongest partnership existed between China and the United States. Conversely, China exhibited limited collaborative ties with other nations. The United States maintained extensive global collaborative networks. These American connections were particularly prominent with European countries. European nations themselves formed distinct regional research clusters. This clustering likely stems from geographical proximity and supportive European Union research policies. In contrast, research output from African countries remained notably scarce.

### 3.3 Institutional analysis

**Figure 4:**
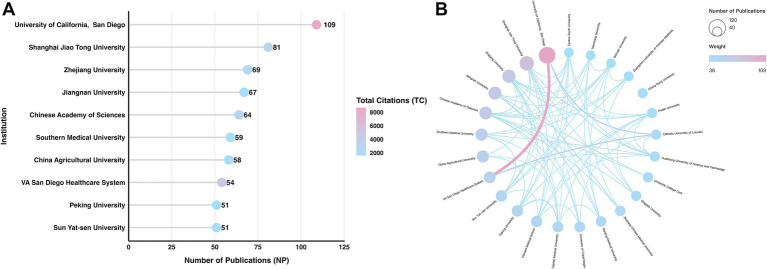
Institutional output and collaboration analysis: **(A)** Top productive institutions and **(B)** institutional collaboration network.

Figure 4A illustrates the top 10 institutions by publication volume and citation impact. In this visualization, redder hues indicate higher citation frequencies. Table 2 details the specific metrics. The University of California, San Diego (UCSD) ranked first in productivity (NP = 109). Shanghai Jiao Tong University (NP = 81) and Zhejiang University (NP = 69) followed. UCSD also maintained an overwhelming lead in total citations (TC = 8,648). Conversely, China Agricultural University published 58 articles but recorded a total link strength (TLS) of only 5. This unusually low TLS indicates a distinct lack of inter-institutional collaboration.

**Table 2:** Top 10 most productive institutions.

Rank	Institution	NP	TC	TLS
1	University of California, San Diego	109	8,648	102
2	Shanghai Jiao Tong University	81	2,768	31
3	Zhejiang University	69	2,443	32
4	Jiangnan University	67	1,718	18
5	Chinese Academy of Sciences	64	2,793	31
6	Southern Medical University	59	1,620	22
7	China Agricultural University	58	1,960	5
8	VA San Diego Healthcare System	54	4,500	84
9	Peking University	51	1,791	27
10	Sun Yat-sen University	51	1,944	22

NP, number of publications; TC, total citations; TLS, total link strength.

Figure 4B maps the collaborative networks among these institutions. Thicker and redder connecting lines denote stronger partnerships. The closest individual collaboration occurred between UCSD and the VA San Diego Healthcare System. Together with the Catholic University of Louvain, these two institutions formed a tightly integrated collaborative triangle.

### 3.4 Journal analysis

**Figure 5:**
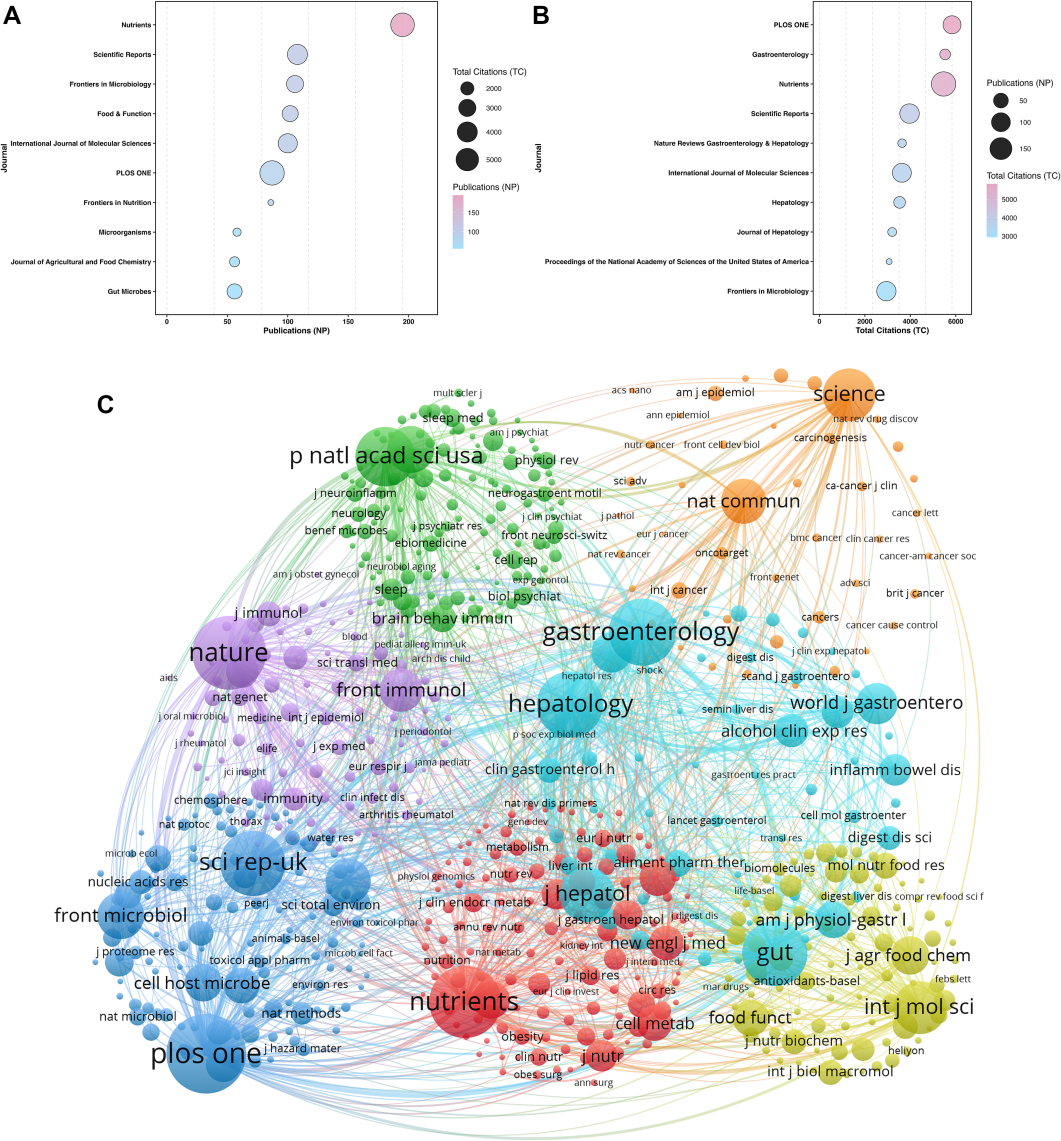
Journal output, citation impact, and co-citation network. **(A)** Top productive journals. **(B)** Top cited journals. **(C)** Node visualization of the journal co-citation network.

Figure 5A maps the top ten most productive journals in this domain. Bubble size corresponds to total citations. Color intensity reflects the number of publications. Table 3 details these comprehensive metrics. *Nutrients* (IF = 5.0) dominates the publication volume (NP = 195). *Scientific Reports* (IF = 3.9; NP = 108) and *Frontiers in Microbiology* (IF = 4.5; NP = 106) drive secondary output. These specific platforms constitute the primary academic dissemination channels.

**Table 3:** Top 10 most productive journals.

Rank	Journal	NP	TC	IF (2024)	JCR (2024)
1	Nutrients	195	5,463	5.0	Q1
2	Scientific Reports	108	3,965	3.9	Q1
3	Frontiers in Microbiology	106	2,946	4.5	Q1
4	Food & Function	102	2,653	5.4	Q1
5	International Journal of Molecular Sciences	100	3,628	4.9	Q1
6	PLOS ONE	87	5,835	2.6	Q2
7	Frontiers in Nutrition	86	1,260	5.1	Q1
8	Microorganisms	58	1,334	4.2	Q2
9	Gut Microbes	56	2,411	11.0	Q1
10	Journal of Agricultural and Food Chemistry	56	1,496	6.2	Q1

NP, number of publications; TC, total citations; IF (2024), 2024 impact factor; JCR, Journal Citation Reports quartile.

Figure 5B delineates the top ten most cited journals. Bubble size denotes NP. Color intensity represents TC. Table 4 summarizes these core impact metrics. *PLOS ONE* (IF = 2.6) ranks sixth in absolute output (NP = 87) but commands the highest overall impact (TC = 5,835). *Gastroenterology* (TC = 5,532) and *Nutrients* (TC = 5,463) maintain comparable high citation volumes. Premier clinical and hepatology specialty journals display an inverse volume-to-impact ratio. Low publication outputs generate disproportionately high citation rates. Specifically, *Gastroenterology* (IF = 25.9; TC = 5,532), *Nature Reviews Gastroenterology & Hepatology* (IF = 51.6; TC = 3,638), *Hepatology* (IF = 15.8; TC = 3,529), and the *Journal of Hepatology* (IF = 33.0; TC = 3,202) anchor the top ten citation rankings.

**Table 4:** Top 10 most cited journals.

Rank	Journal	TC	NP	IF (2024)	JCR (2024)
1	PLOS ONE	5,835	87	2.6	Q2
2	Gastroenterology	5,532	18	25.9	Q1
3	Nutrients	5,463	195	5.0	Q1
4	Scientific Reports	3,965	108	3.9	Q1
5	Nature Reviews Gastroenterology & Hepatology	3,638	9	51.6	Q1
6	International Journal of Molecular Sciences	3,628	100	4.9	Q1
7	Hepatology	3,529	24	15.8	Q1
8	Journal of Hepatology	3,202	10	33.0	Q1
9	Proceedings of the National Academy of Sciences of the United States of America	3,069	5	9.1	Q1
10	Frontiers in Microbiology	2,946	106	4.5	Q1

TC, total citations; NP, number of publications; IF, impact factor; JCR, Journal Citation Reports quartile.

The journal co-citation network (Figure 5C) identifies *PLOS ONE*, *Gastroenterology*, and *Hepatology* as central structural hubs. The core positioning of these premier clinical journals reflects a definitive shift in the research paradigm. Investigations of lifestyle-induced microbial dysbiosis have transitioned from descriptive observations to mature, mechanistic clinical science. Dense co-citation linkages between these journal clusters function as conceptual bridges. They directly integrate systemic metabolic pathway analyses with localized gut microecological alterations. This bibliometric architecture confirms the rigorous clinical foundation of this domain.

### 3.5 Author analysis

**Figure 6:**
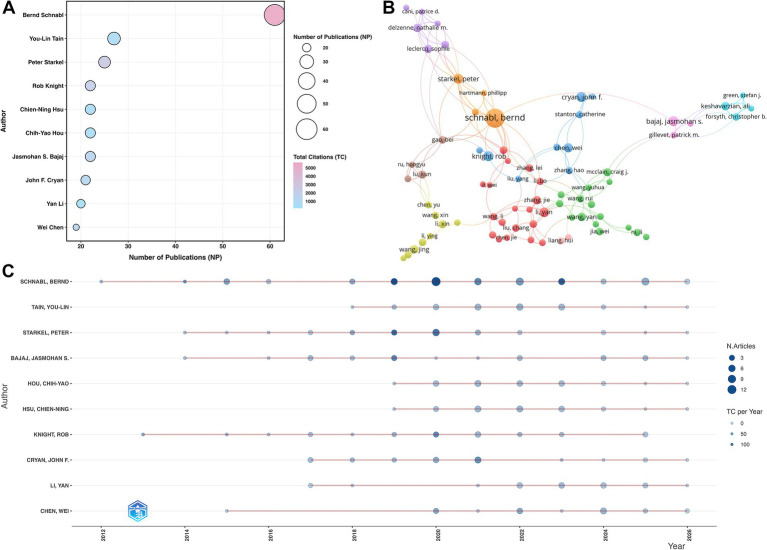
Author productivity, co-occurrence, and annual publication dynamics. **(A)** Top productive authors. **(B)** Node visualization of the author co-occurrence network. **(C)** Annual productivity distribution of the top 10 most productive authors.

Figure 6A maps the top 10 most productive authors. In this visualization, bubble size denotes publication volume, and red color intensity reflects total citations. Table 5 details the specific metrics. Bernd Schnabl ranked first in productivity (NP = 61). You-Lin Tain (NP = 27) and Peter Starkel (NP = 25) followed. Schnabl also held a dominant lead in total citations (TC = 5,637). John F. Cryan presented a distinct academic profile. He published 21 articles but achieved a breakaway H-index of 162. In stark contrast, Yan Li recorded the lowest H-index of 7 among this top cohort.

**Table 5:** Top 10 most productive authors.

Rank	Author	NP	TC	H-index
1	Bernd Schnabl	61	5,637	87
2	You-Lin Tain	27	694	54
3	Peter Starkel	25	2,573	52
4	Jasmohan S Bajaj	22	1,461	98
5	Chih-Yao Hou	22	523	37
6	Chien-Ning Hsu	22	528	48
7	Rob Knight	22	1,977	16
8	John F Cryan	21	1,163	162
9	Yan Li	20	196	7
10	Wei Chen	19	934	31

NP, number of publications; TC, total citations.

Figure 6B visualizes the author co-occurrence network. Researchers in this field formed distinct collaborative clusters. Strong inter-cluster connections were evident across the network. Figure 6C tracks the annual output of these high-yield authors. Their collective publication volume concentrated heavily between 2019 and 2023. Bernd Schnabl demonstrated the most sustained contribution. He maintained continuous output from 2012 to 2025. Following the overall field trend, his personal production also peaked during the 2019–2023 timeframe.

### 3.6 Cited reference analysis

**Figure 7:**
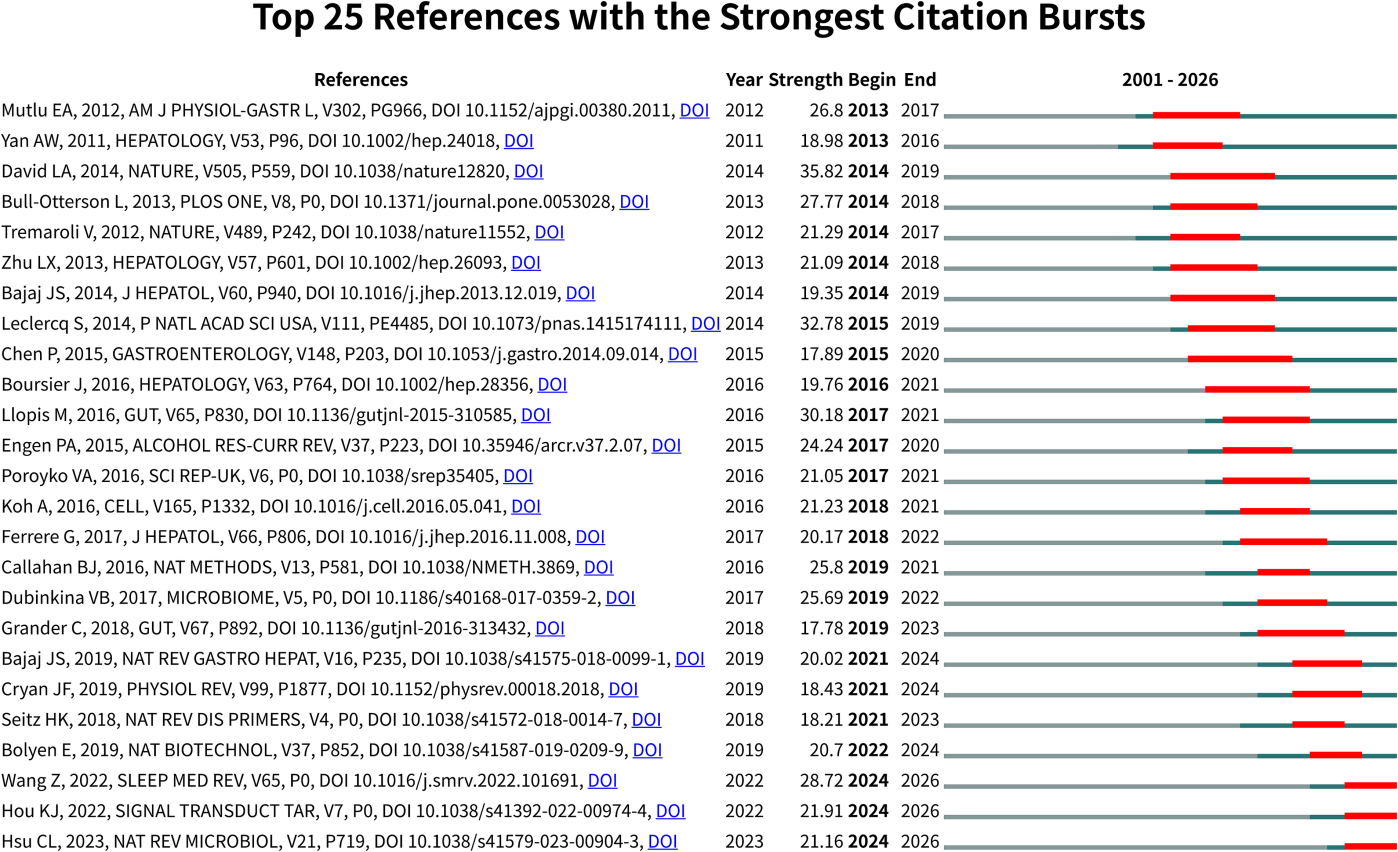
Citation burst analysis of the top 25 references.

Figure 7 maps the top 25 references with the strongest citation bursts utilizing CiteSpace. These citation bursts identify highly impactful milestones shaping the global research agenda. Thematic priorities evolve chronologically. High-impact publications drive these chronological shifts. David et al. (26) published “Diet rapidly and reproducibly alters the human gut microbiome” in *Nature*. This landmark article recorded the highest overall burst strength at 35.82. It established a fundamental causal relationship between dietary exposures and immediate microbial alterations. Similarly, the foundational mapping provided by Huttenhower and the Human Microbiome Project Consortium catalyzed the global transition toward investigating the gut-brain axis and environmental exposures. Leclercq et al. (27) authored “Intestinal permeability, gut-bacterial dysbiosis, and behavioral markers of alcohol-dependence severity” (strength: 32.89). Llopis et al. (28) published “Intestinal microbiota contributes to individual susceptibility to alcoholic liver disease” (strength: 30.18). Both foundational studies significantly advanced the mechanistic understanding of intestinal barrier integrity and alcohol-induced dysbiosis.

The recent timeframe (2022–2026) exhibits a definitive shift toward precision health and systemic metabolic pathways. Wang et al. (29) published highly cited work in *Sleep Medicine Reviews* (strength: 28.72). This publication reflects a major thematic pivot toward the gut-brain axis and sleep-related microbial alterations. Sleep disorders currently dominate the emerging research frontier. This specific trajectory directly corroborates the sub-theme evolution mapped in Figure 2B. Hou et al. (16) (strength: 21.91) and Hsu et al. (30) (strength: 21.16) generated sustained scientific interest. Their publications explicitly investigate the molecular signaling pathways translating unhealthy lifestyle exposures into chronic systemic pathologies.

High burst strengths span the entire chronological timeline. Early foundational milestones include Mutlu et al. (31) (strength: 26.8). Recent systemic evidence features Wang et al. (29) (strength: 28.72). This continuous high-intensity citation pattern indicates rapid domain maturation. The evolutionary trajectory confirms a distinct paradigm shift. Current research strictly prioritizes high-rigor, causal inference models. These advanced investigations definitively link specific lifestyle triggers to profound microbial and systemic pathological outcomes.

### 3.7 Keyword analysis and hotspot evolution

**Figure 8:**
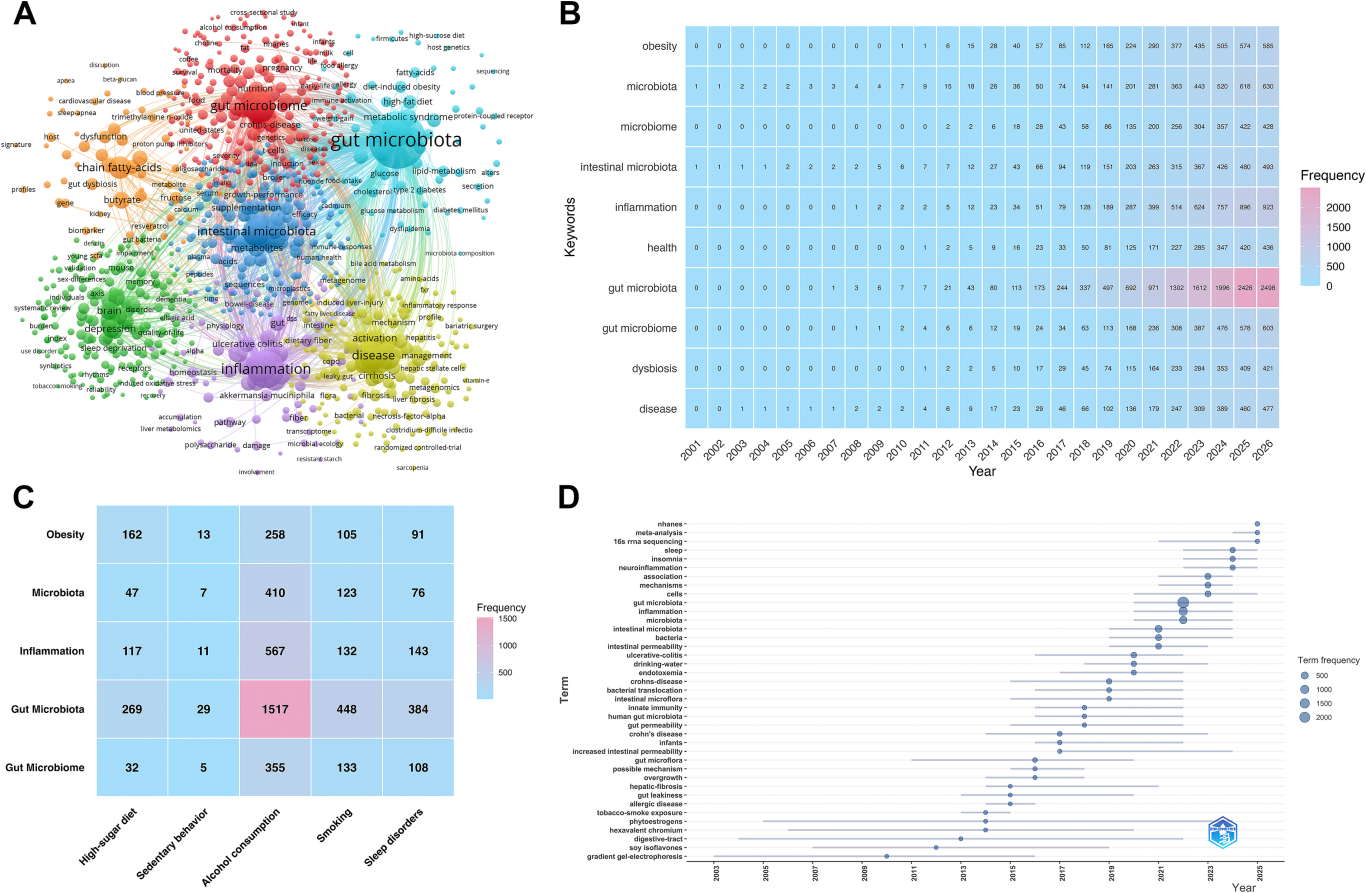
Keyword analysis of the primary domain and cross-analysis of sub-themes. **(A)** Node visualization of the keyword co-occurrence network in the primary domain. **(B)** Density heatmap of keywords in the primary domain. **(C)** Cross-sectional heatmap of keywords across the sub-themes. **(D)** Chronological timeline of thematic trend distribution in the primary domain.

Keyword analysis delineates research hotspots and developmental trajectories. Figure 8A maps the keyword co-occurrence network. The algorithm identified seven distinct clusters. The bibliometrix package extracted the top 20 most frequent keywords (Table 6). Each term appeared over 250 times. “Gut microbiota” recorded the highest frequency (*n* = 2,498). Other leading terms included “inflammation” (*n* = 923), “microbiota” (*n* = 630), “gut microbiome” (*n* = 603), and “obesity” (*n* = 585). All these core terms exceeded 500 occurrences. Beyond the microbiota itself, research heavily targets specific mechanistic pathways. These include inflammation, obesity, oxidative stress, and overarching metabolism. Figure 8B presents a heatmap of the top 10 keywords. Redder hues denote higher frequencies. Microbiota-related terms exhibit rapid, ongoing growth. Concurrently, “inflammation” and “obesity” command increasing investigative focus. Terms such as “gut microbiota,” “microbiota,” and “gut microbiome” are functionally synonymous. We analyzed them independently rather than merging them. This methodological choice accurately reflects the chronological evolution in terminology preferences among researchers.

**Table 6:** Top 20 most frequent keywords.

Rank	Keyword	Frequency
1	gut microbiota	2,498
2	inflammation	923
3	microbiota	630
4	gut microbiome	603
5	obesity	585
6	intestinal microbiota	493
7	disease	477
8	health	436
9	microbiome	428
10	dysbiosis	421
11	oxidative stress	420
12	diet	385
13	alcohol	371
14	metabolism	366
15	risk	361
16	probiotics	337
17	mice	313
18	chain fatty-acids	297
19	expression	284
20	bacteria	252

Subsequent analysis isolated the keywords specific to each of the five unhealthy lifestyles. The highest-frequency keywords from these sub-fields were aggregated. Figure 8C identifies five shared core keywords. “Gut microbiota” appeared universally across all domains. The distribution of the other shared terms matched the patterns in Figure 8B. Among the five factors, “alcohol consumption” yielded the highest volume of associated keywords. This volume aligns with the thematic publication distribution shown in Figure 2B.

Figure 8D illustrates the chronological evolution of thematic keywords. The field exhibited distinct developmental phases. Research prior to 2015 yielded sparse keyword occurrences. Early terms included “gradient gel-electrophoresis” (2010), “soy isoflavones” (2012), and “phytoestrogens” (2014). “Tobacco-smoke exposure” emerged as a frequent keyword in 2014. By 2017, “Crohn’s disease” appeared as a primary disease-related term. Keyword frequencies increased rapidly between 2018 and 2022. High-frequency terms during this period included “gut permeability” (2018), “endotoxemia” (2019), “bacterial translocation” (2019), and “ulcerative colitis” (2020). By 2022, outputs concentrated heavily on “gut microbiota,” “inflammation,” and “bacteria.” This concentration matches the annual sub-theme publication trends presented in Figure 2B. From 2023 onward, the thematic focus shifted. The year 2024 showed a surge in terms such as “sleep,” “insomnia,” and “neuroinflammation.” This emergence aligns with the increased volume of sleep disorder studies noted in Figure 2B. By 2025, methodological and epidemiological terms became prominent. Coinciding with the broader adoption of foundational technologies and public databases, “16S rRNA sequencing,” “nhanes,” and “meta-analysis” emerged as dominant keywords in 2025.

**Figure 9:**
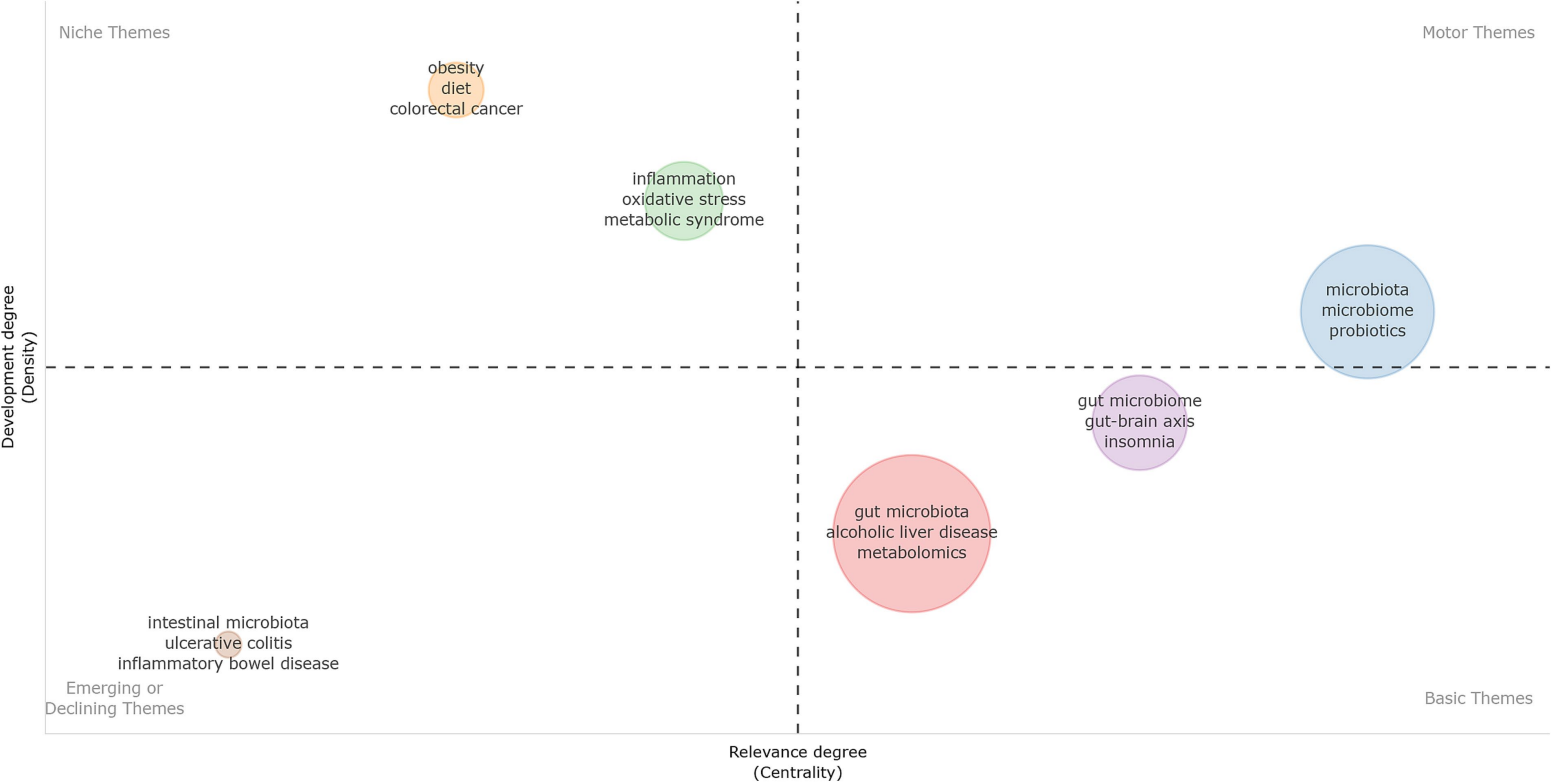
Thematic landscape map of research concerning the impact of unhealthy lifestyles on the gut microbiota.

Figure 9 presents a Thematic Map synthesizing the collective keywords across all five unhealthy behaviors. This map plots clusters based on their centrality (relevance) and density (development). The Motor Themes quadrant (high density, high centrality) represents well-developed and core research topics. This upper-right quadrant features the “microbiota/microbiome” and “probiotics clusters.” The Basic and Transversal Themes quadrant (low density, high centrality) captures foundational topics. Here, large clusters for “alcoholic liver disease” and “metabolomics” dominate the horizontal axis. Additionally, a distinct cluster containing “gut-brain axis” and “insomnia” is positioned in this lower-right quadrant. Its topological position is rapidly advancing toward higher centrality regions, indicating increasing co-occurrence with diverse research domains. The Niche Themes quadrant (high density, low centrality) displays highly specialized but isolated topics. Clusters including “obesity,” “diet,” and “colorectal cancer” reside in this upper-left quadrant, forming internally mature, self-contained bibliometric networks. Furthermore, clusters for “inflammation,” “oxidative stress,” and “metabolic syndrome” are situated strictly at the border between the first and second quadrants. Topologically, this border placement acts as a structural bridge, linking highly specialized topics to the core network dynamics. Finally, the Emerging or Declining Themes quadrant (low density, low centrality) includes the “inflammatory bowel disease” and “ulcerative colitis” clusters. Correlating with the timeline in Figure 8D, these specific terms currently exhibit lower relative momentum within the overall thematic network.

### 3.8 Clinical research analysis

**Figure 10:**
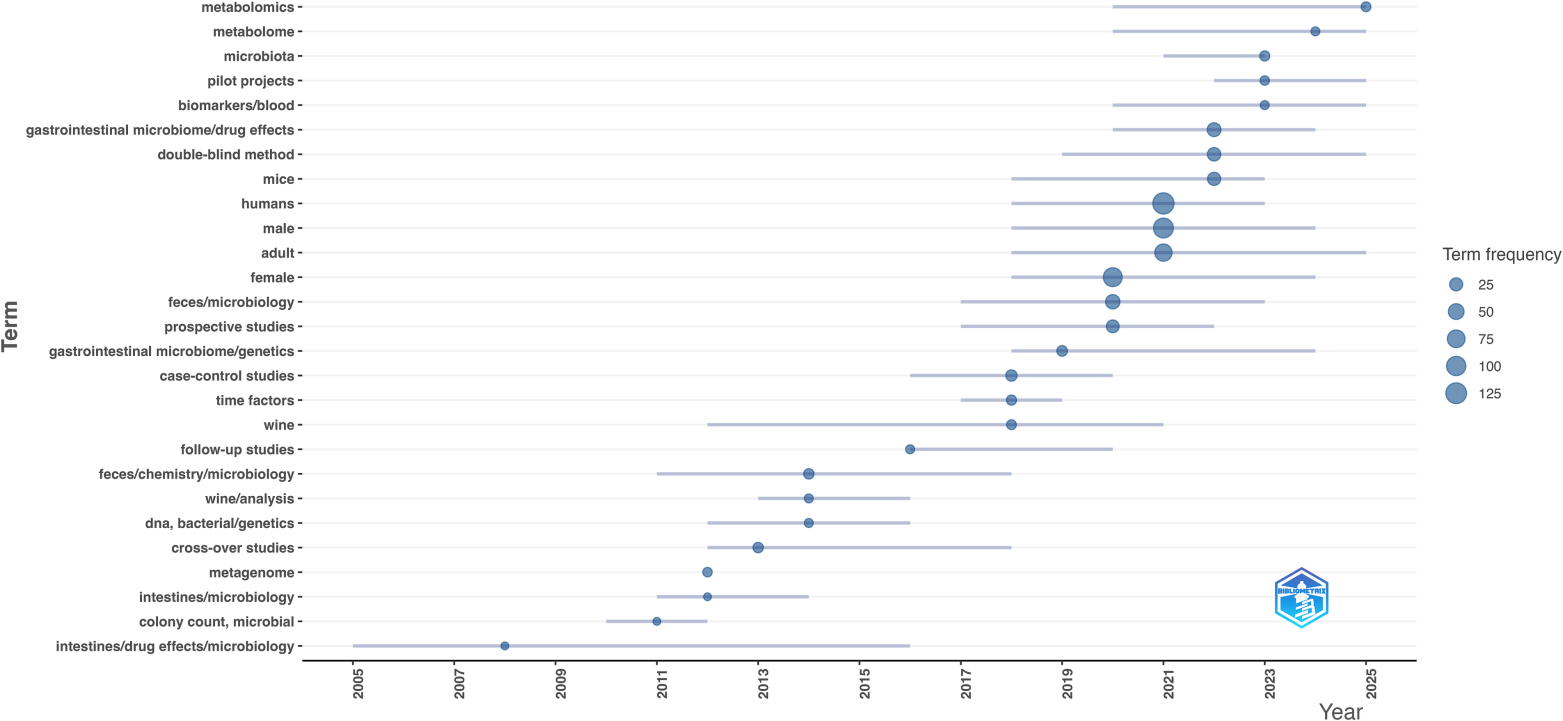
Chronological timeline of thematic trend distribution in clinical research.

To explicitly evaluate clinical translation within this field, we analyzed the dedicated PubMed dataset. Figure 10 illustrates the chronological evolution of these clinical hotspots. Keywords such as “wine/analysis” (2014) and “wine” (2016) emerged as initial focal points. These occurrences confirm that alcohol consumption attracted early clinical attention. Between 2018 and 2021, the research focus shifted toward defined human demographics and applied clinical designs. Prominent demographic keywords included “case–control studies” (2018), “female” (2020), “prospective studies” (2020), “humans” (2021), “adult” (2021), and “male” (2021). The emergence of these terms indicates a clear transition toward distinct human population studies.

From 2022 onward, clinical research increasingly integrated systemic multi-omics approaches. High-frequency terms during this recent phase included “biomarkers/blood” (2022), “double-blind method” (2022), “microbiota” (2023), “pilot projects” (2023), “metabolome” (2024), and “metabolomics” (2025). This keyword convergence reflects a specific methodological trajectory. Recent studies increasingly utilize blood biomarkers and metabolomic profiling to establish systemic connections with the gut microbiota.

Overall, thematic trends reveal a clear progression in clinical trial rigor. Methodological terms evolved sequentially across the study period. They progressed from early “cross-over studies” (2013) to “prospective studies” (2020), and most recently to the “double-blind method” (2022). This chronological trajectory indicates a continuous enhancement in the methodological design of clinical investigations.

## 4 Discussion

### 4.1 General overview and trajectories

This study quantified the research landscape concerning the impact of unhealthy lifestyles on the gut microbiota. Standard bibliometric and visualization methods were applied. Two independent databases were utilized. The primary dataset comprised 5,380 WoSCC articles. A parallel clinical analysis utilized 172 PubMed articles. Overall publication volumes exhibited substantial growth from 2001 to 2026. Data collection for 2026 was restricted to a three-month period. Despite this partial dataset, the fitted trend line projects continued significant growth for the full year.

The publication trajectory reflects three distinct developmental eras, driven by technological and historical milestones. Research output during the initial phase (2001–2011) remained sparse and fluctuating. In this decade, high-throughput sequencing technologies were prohibitively expensive and methodologically nascent (32). This critical technological bottleneck strictly limited large-scale microbiome profiling. The subsequent steady expansion (2012–2018) aligns directly with major scientific breakthroughs. In 2012, the Human Microbiome Project published its landmark phase one results (33). This initiative shifted gut microecology into mainstream clinical science. It served as a crucial catalyst, directing global academic focus toward environmental and lifestyle exposures. The recent exponential surge (2019–2025) stems from both methodological and epidemiological drivers (34). Methodologically, this period witnessed the maturation of multi-omics integration. Epidemiologically, the COVID-19 pandemic necessitated unprecedented lockdowns and social isolation. These pandemic measures drastically exacerbated sedentary behaviors, alcohol abuse, and poor dietary habits worldwide (35). The field exhibited profound resilience against institutional disruptions. Consequently, this widespread deterioration in population lifestyle inadvertently provided massive real-world cohorts and powerful epidemiological momentum for this research domain.

China currently leads global publication output. This dominance stems from an extensive domestic research workforce. It is also heavily propelled by national strategic initiatives, specifically the “Healthy China 2030” blueprint (36). Lifestyle-microbiome research offers immense preventive public health value for China’s rapidly aging population (37). Translating these findings into dietary and behavioral interventions directly reduces national healthcare expenditures and elevates the overall quality of life. The United States follows closely. The high national prevalence of the Standard American Diet (SAD) and sedentary behavior drives urgent epidemiological interest. Sustained funding from the National Institutes of Health (NIH) for chronic disease prevention strongly supports this American output (38). European nations formed distinct collaborative research clusters. This regional integration is largely driven by geographic proximity and structured funding frameworks like “Horizon Europe” (39).

Institutional analysis highlights distinct global leaders. UCSD, Shanghai Jiao Tong University, and Zhejiang University emerged as primary academic producers. UCSD specifically anchors the global network. UCSD, the VA San Diego Healthcare System, and the Catholic University of Louvain form a tightly integrated collaborative triad. While Chinese institutions generate high publication volumes, they exhibit weaker international linkages. Chinese research centers must actively integrate into global collaborative networks. Cross-continental collaboration is essential to overcome population-specific microbiome biases and validate findings across diverse genetic and dietary backgrounds (40).

Publication metrics reflect high academic rigor and profound clinical relevance within this field. *Nutrients* (IF = 5.0) dominates the absolute volume with 195 publications and 5,463 total citations. Core scientific journals, including *Scientific Reports* (IF = 3.9; NP = 108; TC = 3,965) and *Frontiers in Microbiology* (IF = 4.5; NP = 106; TC = 2,946), drive consistent output. Citation impact metrics reveal profound clinical gravity. Nine of the top 10 most-cited journals rank in JCR Q1. The mega-journal *PLOS ONE* (IF = 2.6) achieved 5,835 citations. Crucially, leading medical and hepatology journals actively publish in this domain. These include *Gastroenterology* (IF = 25.9; TC = 5,532), *Nature Reviews Gastroenterology & Hepatology* (IF = 51.6; TC = 3,638), *Hepatology* (IF = 15.8; TC = 3,529), and the *Journal of Hepatology* (IF = 33.0; TC = 3,202). The continuous involvement of these premier clinical journals, supported by a dense and active author collaboration network, firmly validates the rigorous academic standing of this research field.

### 4.2 Research hotspots and methodological evolution

Comprehensive bibliometric visualization delineates current research hotspots and emerging priorities regarding lifestyle-microbiome interactions. Citation analysis highlights highly impactful foundational works. Hou et al. (16) established the gut microbiota as a central mediator of health and disease. Their work mechanistically linked multiple unhealthy lifestyles to the gut-brain axis, colonization resistance, and immune regulation. Recent work by Hsu et al. (30) reinforces the profound necessity of healthy lifestyles for maintaining microbial homeostasis. Researchers no longer restrict their focus to isolated, microscopic mechanisms. Broad public health implications and macroscopic epidemiological impacts now dominate the research agenda. Keyword analysis confirms a persistent thematic core. Investigations consistently target the gut microbiota and associated metabolomics across all timeframes. Thematic trends also reveal distinct chronological shifts in specific exposures. Alcohol consumption attracted the earliest extensive clinical attention. Recently, the research frontier transitioned sharply toward sleep disorders. Keywords including “sleep,” “insomnia,” and “neuroinflammation” surged dramatically in 2024.

Methodological paradigms exhibit a parallel evolution. Technological constraints heavily restricted early investigations. Prior studies relied on basic techniques like gradient gel electrophoresis and focused narrowly on traditional pathologies like inflammatory bowel disease (41). Subsequent technological advancements revolutionized this landscape. Current bench methodologies heavily prioritize 16S rRNA sequencing. Concurrently, computational researchers increasingly leverage massive public databases and systematic reviews (42). Keywords like “nhanes” and “meta-analysis” emerged as dominant recent trends. This specific methodological shift warrants critical appraisal. Over-reliance on easily accessible public databases presents severe scientific limitations. These datasets have inadvertently catalyzed a proliferation of low-quality, derivative publications. This massive influx obscures genuine scientific hotspots and generates substantial background noise (43). It places immense strain on the global peer-review infrastructure and dilutes the overall quality of evidence synthesis. Consequently, the field urgently demands rigorous causal inference rather than mere correlational data mining. Advanced genetic epidemiology techniques, specifically Mendelian Randomization (MR), are rapidly gaining prominence (44). Researchers now increasingly utilize MR to definitively establish unidirectional causal relationships between lifestyle exposures and specific microbial alterations.

#### 4.2.1 Associations between unhealthy lifestyles, inflammation, oxidative stress, and gut microbiota

Keyword and cross-domain analyses identify inflammation as the primary mechanistic hotspot. Thematic mapping positions “inflammation,” “oxidative stress,” and “metabolic syndrome” as vital structural bridges. These mechanisms dynamically link core microbiome research to highly specialized phenotypes like the gut-brain axis and insomnia.

Tobacco smoke introduces potent toxins, including nicotine, into the systemic circulation. Nicotine has been associated with impaired intestinal mucosal barrier integrity (45). This barrier dysfunction permits luminal antigen infiltration. The host immune system mounts a robust local oxidative stress response, which is associated with microbial dysbiosis. Alcohol exerts direct and immediate luminal toxicity. Ethanol metabolism generates excessive reactive oxygen species and acetaldehyde. This localized oxidative stress is associated with the degradation of epithelial tight junction proteins (46). Subsequent endotoxin translocation is associated with heightened immune signaling, which may contribute to gastrointestinal inflammation and alterations in microbial architecture. High-sugar diets inflict chronic, cumulative microbiome disruption (47). Recent meta-analyses demonstrate that sustained high-sucrose intake systematically starves short-chain fatty acid (SCFA)-producing taxa. This critical SCFA depletion impairs mucosal immune tolerance. It establishes a persistent, low-grade inflammatory microenvironment hostile to commensal bacteria (48). Sleep deprivation represents a critical emerging frontier governed by the gut-brain axis. Sleep disruption fundamentally dysregulates neuroendocrine pathways (49). Specifically, psychological stress and sleep deprivation hyperactivate the hypothalamo-pituitary–adrenal (HPA) axis, functioning as a primary bidirectional conduit between the brain and the gut microenvironment. It specifically suppresses anti-inflammatory melatonin and drives cortisol hypersecretion. Animal models confirm this specific hormone imbalance rapidly accelerates systemic and gastrointestinal inflammation, directly altering gut microbial composition and intestinal barrier integrity (50). Combined public database and Mendelian Randomization analyses corroborate this phenomenon. They demonstrate acute oxidative stress elevation directly following sleep restriction (51). The dramatic 2024 keyword surge for “sleep,” “insomnia,” and “neuroinflammation” directly reflects this pathogenic reality. Prolonged sedentary behavior dominates modern high-intensity occupational and academic environments. Chronic physical inactivity mechanically slows gastrointestinal transit time (52). Comprehensive meta-analyses reveal that delayed intestinal transit directly suppresses overall microbial diversity. It simultaneously fosters the overgrowth of pro-inflammatory pathobionts (53).

Inflammation and oxidative stress are not only associated with microbial dysbiosis but also represent central and unifying biological pathways linking diverse unhealthy lifestyle exposures with alterations in the gut microbiome (54).

#### 4.2.2 Obesity and metabolic dysfunction serve as the critical bridge

Keyword frequencies and heatmaps identify “obesity” and “metabolism” as primary research hotspots. Thematic mapping positions “obesity” within the Niche Themes quadrant (high density, low centrality). This placement indicates a highly specialized, internally mature, and self-contained bibliometric network. “Alcoholic liver disease” and “metabolomics” occupy foundational, core positions within the overall research landscape. Obesity represents the most prevalent macroscopic consequence of unhealthy lifestyles (55).

As the most ubiquitous phenotypic manifestation of unhealthy lifestyles, obesity currently burdens over one billion individuals globally (56). Diverse maladaptive behaviors convergently drive this metabolic crisis. Crucially, the trajectory toward obesity and concurrent insulin resistance is intimately associated with profound compositional shifts in the gut microbiota—most notably, an elevated Firmicutes-to-Bacteroidetes ratio and a severe depletion of butyrate-producing commensals (57). This microbial dysbiosis is not merely an epiphenomenon; it actively compromises glucose homeostasis and perpetuates systemic low-grade inflammation. Consequently, it serves as a core pathogenic bridge linking lifestyle exposures to an array of metabolic and endocrine disorders, including type 2 diabetes (58). Reinforcing this exposure-to-phenotype continuum, recent Mendelian randomization (MR) analyses have further established a definitive causal pathway between specific behavioral triggers, such as excessive alcohol consumption, and increased adiposity (59).

Ethanol processing monopolizes hepatic metabolism. This prioritization actively halts lipid oxidation and accelerates *de novo* lipogenesis. It drives rapid visceral fat accumulation (60). Sleep disorders exhibit strong epidemiological correlations with obesity in massive public database analyses (61). Animal models validate this causal direction (62). Sleep disruption skews the ghrelin-to-leptin ratio. This hormonal imbalance induces hyperphagia and subsequent rapid weight gain (63). Early keyword hotspots included “phytoestrogens” in 2014. This reflects initial attempts to hormonally modulate metabolic dysfunction. Smoking acts as a severe endocrine disruptor. It specifically drives central visceral adiposity and profound insulin resistance. It acts as a primary catalyst for systemic metabolic collapse. High-sugar diets and sedentary behaviors serve as the most direct drivers of this metabolic crisis. Sustained sucrose intake overwhelms hepatic metabolic capacity. It triggers rapid insulin resistance and profound adipocyte hypertrophy. Prolonged sedentary behavior drastically reduces skeletal muscle glucose clearance. This physical inactivity directly initiates systemic lipid accumulation.

Hou et al. (16) reported that the resulting metabolic dysfunction may act as a primary pathogenic mechanism and is associated with gut microbiome dysbiosis. The obese physiological state exponentially amplifies systemic organ burden. It multiplies the probability of cascading chronic metabolic disorders (64). Ultimately, obesity and metabolic dysfunction are closely associated with both discrete behavioral exposures and chronic systemic alterations in gut microecology, and are considered important biological intermediates in this process.

#### 4.2.3 Preventive strategies to mitigate microbiome dysbiosis

Unhealthy behavioral patterns fundamentally drive gut microbiota dysbiosis (65). Modern occupational and academic demands systematically elevate chronic psychological stress (66). Excessive caffeine intake and prolonged electronic screen exposure actively disrupt circadian rhythms (67). These environmental stressors make sleep disorders increasingly ubiquitous. Our keyword analysis directly identifies “sleep” and “insomnia” as primary pathogenic drivers. Preventive public health strategies must prioritize rigorous sleep hygiene. Restoring optimal sleep architecture represents a mandatory non-pharmacological intervention to prevent neuroinflammatory dysbiosis.

The globalization of the beverage industry and advanced distribution networks have drastically increased global alcohol accessibility. “Alcohol consumption” represents a dominant and escalating epidemiological threat. Thematic trend analysis explicitly links excessive alcohol intake to the exacerbation of severe gastrointestinal pathologies, including “ulcerative colitis” and “Crohn’s disease.” Previous controversial studies suggested potential cardiovascular benefits from moderate alcohol consumption. Current global health consensus definitively refutes this paradigm. The WHO explicitly states that no level of alcohol consumption is safe for human health (68). Strict abstinence is mandatory to prevent “alcoholic liver disease” and severe microbiome degradation.

Tobacco use remains a primary global health hazard. Our bibliometric data identified “phytoestrogens” (2014) as an early research hotspot. This reflects the initial recognition of environmental endocrine disruptors. Tobacco smoke functions as a potent, microbiome-altering endocrine disruptor. Public health mandates must aggressively target both active smoking and passive secondhand smoke exposure. Complete tobacco cessation is critical to maintain intestinal mucosal barrier integrity (17).

High-sugar diets exploit fundamental human neurobiology. Dietary sucrose triggers rapid dopamine release in the mesolimbic system. This neurochemical reinforcement induces strong reward-seeking behaviors and transient euphoria. This dietary pattern directly drives catastrophic obesity and metabolic dysfunction. Current Western dietary demographics reflect extreme overconsumption. For instance, the average American consumes approximately 17 teaspoons of added sugar daily (69). This volume vastly exceeds the strict limits recommended by the American Heart Association. Public health campaigns must actively educate populations on hidden dietary sugars. Strict regulation of high-sugar diets is essential to prevent metabolic dysbiosis.

Sedentary behavior is endemic in modern industrialized societies. Prolonged physical inactivity fundamentally suppresses gastrointestinal motility and microbiome diversity (70). Preventive strategies must actively disrupt prolonged sitting patterns. Integrating structured, daily physical exercise is essential to stimulate intestinal transit and preserve optimal gut microecology.

### 4.3 Evolution and maturation of clinical translation

The clinical landscape of lifestyle-microbiome research exhibits fundamental maturation. Early microbiome studies faced massive confounding variables. The field has now transitioned from exploratory observations to rigorous causal investigations (71). Moving forward, reliance on traditional 16S rRNA sequencing must give way to advanced high-resolution techniques, including shotgun metagenomics and nutrigenomics, to truly decode individualized metabolic response (72). Furthermore, because baseline gut microecology is highly sensitive to regional diets and genetics, the establishment of population-specific microbiota databases (e.g., Twnbiome) is crucial for developing personalized, lifestyle-targeted interventions (73). The sequential emergence of “prospective studies” and “double-blind methods” directly addresses previous methodological limitations. Current high-quality trials predominantly evaluate targeted “probiotics” and controlled “diet” interventions. This methodological evolution isolates the therapeutic efficacy of behavioral modifications. It definitively establishes causal links between lifestyle interventions and microbiome restoration.

Clinical focus now transcends the gut lumen. The surge in “metabolomics” and “blood biomarkers” signals a profound paradigm shift. Researchers no longer merely profile bacterial taxa. They track microbially derived metabolites entering systemic circulation (74). The frequent keyword “chain fatty-acids” identifies SCFAs as the primary molecular bridge. Unhealthy behaviors critically deplete SCFA-producing bacteria. High-sugar diets and sedentary habits serve as primary drivers of this depletion. Keyword trends indicate that researchers frequently link SCFA deficiency to the onset of “inflammation,” “oxidative stress,” and subsequent “obesity.” Studies utilizing multi-omic integration frequently explore the association between localized “dysbiosis” and systemic pathological cascades. It provides highly actionable targets for clinical diagnostics.

The field increasingly recognizes the necessity of demographic stratification. Early clinical attention heavily targeted “alcohol” due to its obvious gut-liver axis phenotype. Subsequent research broadened to specific demographic populations. Keywords such as “female,” “male,” and “adult” highlight a transition toward precision medicine. Current consensus indicates that biological sex fundamentally dictates microbial responses to lifestyle stressors. Driven by sex hormone interactions, male and female microbiomes exhibit distinct dysbiosis trajectories. They show divergent patterns of metabolic deterioration even under identical unhealthy habits. Future clinical trials must incorporate these sex-specific baselines (75). This biological stratification is essential to develop precisely targeted interventions.

### 4.4 Research significance and implications

This study integrates advanced bibliometrics, custom algorithmic scripting, and diverse visualization technologies. This methodological approach overcomes traditional bibliometric limitations. Dual-database cross-validation delineates the academic trajectories of five prevalent unhealthy lifestyles impacting the gut microbiota. This comprehensive analysis maps specific future research directions. It defines the distinct developmental contours of these five behavioral sub-themes.

The findings highlight key pathophysiological associations. Immunity, inflammation, and oxidative stress are closely associated with both unhealthy behaviors and gut microbial dysbiosis, and are considered important biological mediating factors in this relationship. Cross-domain keyword analyses highlight obesity as a prominent research focus. The literature frequently investigates obesity as a central factor associated with cascading metabolic comorbidities. The bibliometric trends reflect strong academic interest in the consequences of metabolic dysfunction. The literature frequently explores its potential role in linking localized gut dysbiosis to multi-organ pathologies. The chronological analysis captures a parallel methodological shift. It documents the recent proliferation of public database utilization, specifically National Health and Nutrition Examination Survey (NHANES), alongside large-scale systematic meta-analyses.

This study establishes a macroscopic foundation for public health interventions. It offers data-driven strategies for targeted microbiota regulation and gastrointestinal health optimization. Translating these structural insights into actionable behavioral modifications elevates population quality of life. Proactive lifestyle-microbiome management reduces chronic disease incidence. This preventive paradigm systematically decreases long-term healthcare expenditures for governments and medical institutions.

## 5 Limitations

This study presents specific methodological limitations. First, the primary bibliometric dataset relied predominantly on the Web of Science Core Collection. Although supplemented by PubMed, relevant studies indexed in other databases may have been overlooked. Second, the search was restricted to English language publications, which may introduce selection bias and exclude valuable evidence from non-English and regionally indexed sources. Consequently, certain localized or culturally specific representative studies may be underrepresented (76). The scope of this analysis focuses on five predominant unhealthy lifestyles identified based on their substantial epidemiological burden and global health relevance, as informed by the GBD study, WHO, and the American Heart Association’s LE8 framework. However, real-world behavioral exposures are inherently multifactorial and frequently co-occurring. Consequently, our literature retrieval strategy may not have fully captured the complex synergistic interactions among these lifestyle factors. This limitation highlights an important gap and underscores the need for future mechanistic investigations to incorporate a broader spectrum of intersecting behaviors in order to elucidate their combined and potentially synergistic effects on the gut microbiota.

## 6 Conclusion

This study provides a comprehensive bibliometric mapping of research on lifestyle–microbiome interactions from 2001 to 2026. Publication output exhibits sustained, exponential growth. The global COVID-19 pandemic fundamentally accelerated this trajectory. China and the United States lead global research production. Their extensive academic output drives fundamental advancements in public health and gastroenterology. Thematic hotspots center on microbiome profiling and metabolomics. Prominent themes in the literature regarding pathogenic mechanisms include inflammation, oxidative stress, and SCFA depletion. Systemic pathological outcomes encompass obesity, metabolic dysfunction, and emerging gut-brain axis disruptions, specifically sleep disorders. Clinical research demonstrates a decisive evolution toward methodological rigor. Recent clinical trials prioritize double-blind designs, systemic blood biomarkers, and demographic stratification. These findings clarify the complete structural landscape of this field. Future investigations must leverage advanced omics and causal inference models to decode microenvironmental interactions. Deepening this causal understanding yields highly precise, targeted preventive strategies. These lifestyle-based interventions mitigate the global burden of cascading chronic diseases and optimize population health.

## Data Availability

The original contributions presented in the study are included in the article/Supplementary material, further inquiries can be directed to the corresponding authors.
