# Supplementary figures and images for: Associations between prevalent unhealthy lifestyles and the gut microbiota: a comprehensive multi-database bibliometric analysis of pathogenic mechanisms and clinical trajectories
Source: Front Med (Lausanne). 2026 May 12;13:1834916. doi: 10.3389/fmed.2026.1834916 (PMC13201409; doi:10.3389/fmed.2026.1834916)

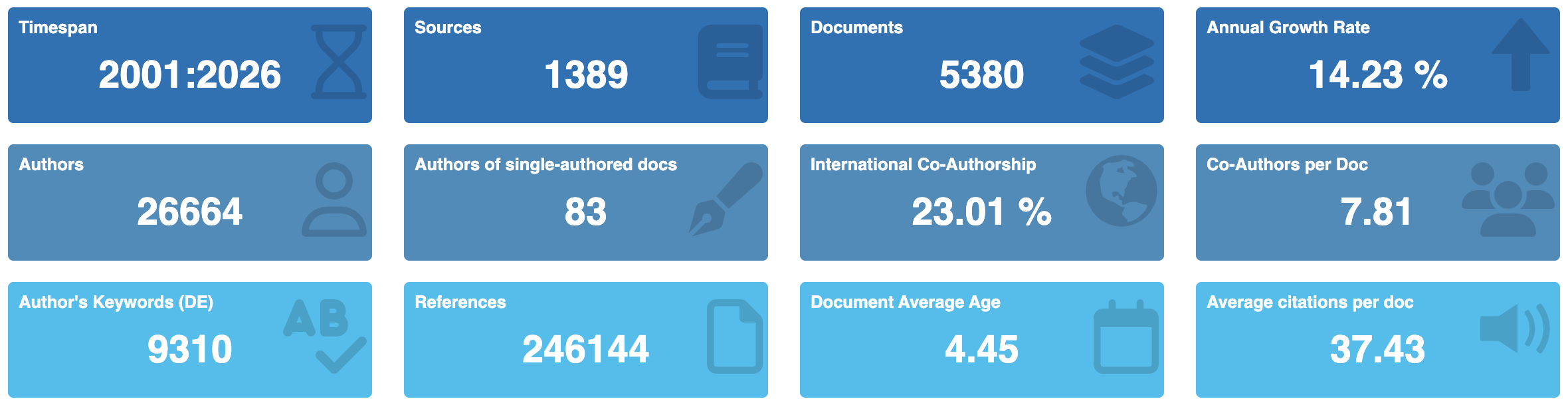

Supplement: Supplementary file 1 [file Image_1.png]

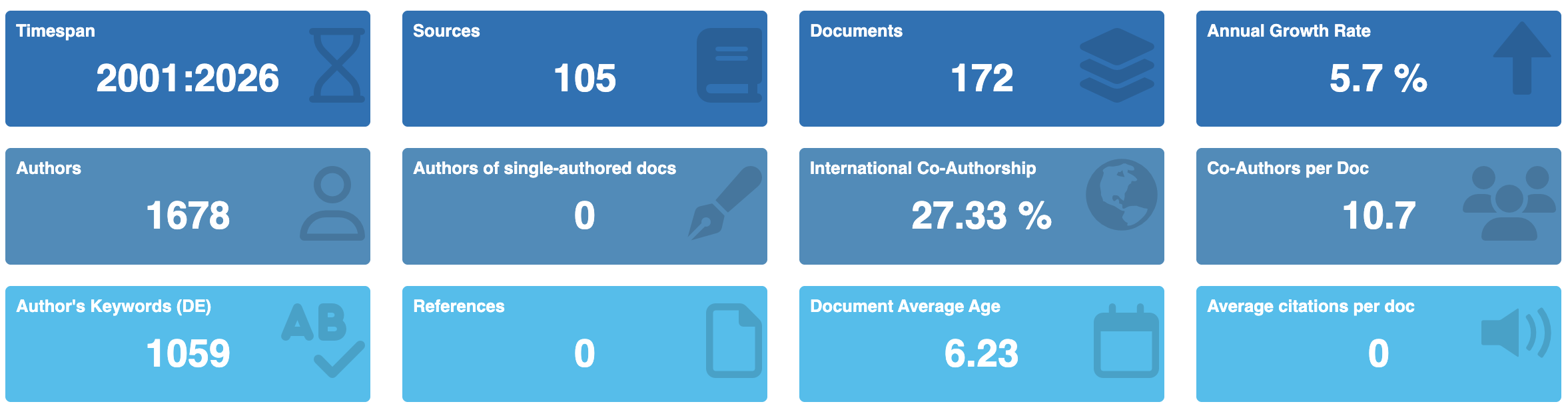

Supplement: Supplementary file 2 [file Image_2.png]
